# Profiling dysregulated circRNA expression in diabetic retinopathy: elucidating putative mediators via a streptozotocin-induced mouse model

**DOI:** 10.3389/fendo.2026.1804009

**Published:** 2026-05-14

**Authors:** Conghui Zhang, Le Feng, Qian Li, Hongping Cui

**Affiliations:** 1 Department of Ophthalmology, Shanghai East Hospital, School of Medicine, Tongji University, Shanghai, China; 2 Department of Ophthalmology, Shanghai Tenth People’s Hospital, Tongji University, Shanghai, China

**Keywords:** circular RNA, diabetic mice, diabetic retinopathy, microRNA, retina, streptozotocin (STZ)

## Abstract

**Background:**

Circular RNAs (circRNAs), a conserved class of non−coding RNAs, are hypothesized to play functional roles in diabetic retinopathy (DR), yet their expression landscape and molecular involvement in retinal tissue remain poorly defined.

**Methods:**

A streptozotocin−induced diabetic mouse model (C57BL/6J, male, n=40) was established, with diabetes confirmed by persistent hyperglycemia (fasting blood glucose >300 mg/dL). Retinal function was assessed by electroretinography (ERG) and vascular pathology by fluorescein fundus angiography (FFA) at 12 weeks post−induction. Retinal circRNA profiles were obtained via microarray analysis. Bioinformatic tools were used to predict circRNA-miRNA interactions, and Gene Ontology (GO) analysis was applied to annotate potential target genes. Differential expression of selected circRNAs was validated by quantitative PCR (qPCR).

**Results:**

Diabetic mice exhibited sustained hyperglycemia, reduced body weight, significant declines in ERG a and b wave amplitudes, and early microangiopathy evident on FFA. Microarray profiling identified 21 dysregulated circRNAs (3 upregulated, 18 downregulated), classified as antisense, exonic, intronic, or intragenic. Bioinformatics reconstruction revealed a circRNA-miRNA network with shared microRNAs (miRNAs), and GO enrichment highlighted processes including transcriptional regulation and cancer−related signaling. qPCR confirmed the expression changes of four circRNAs. Cross−species alignment showed high sequence homology between four murine circRNAs and human orthologs.

**Conclusion:**

This study identifies 21 conserved circRNAs with altered expression in the diabetic retina, supporting their potential role as competitive endogenous RNAs (ceRNAs) and implicating them in DR pathogenesis.

## Introduction

Irreversible visual impairment may be induced by two principal clinical manifestations that drive the progression of DR: diabetic macular edema (DME) and proliferative diabetic retinopathy (PDR) (1). Breakdown of the blood-retinal barrier constitutes the pathological signature of DME, whereas the pathogenesis of both DME and PDR is facilitated by increased levels of vascular endothelial growth factor (VEGF) to a certain extent. In addition to the compromise of the barrier, chronic hyperglycemia appears to elicit a spectrum of pathogenic signaling cascades, which include the activation of protein kinase C (PKC) (2, 3), the accumulation of advanced glycation end-products (AGEs) (4–6), the overproduction of reactive oxygen species (ROS) (7), dysregulated integrin signaling, and heightened concentrations of inflammatory cytokines, with transforming growth factor-β (TGF-β), platelet-derived growth factor (PDGF), and tumor necrosis factor-α (TNF-α) among them (8, 9). Not only do these mechanisms seem to aggravate vascular dysfunction, but they also appear to induce early neuronal injury in DR through signaling cascades that involve nuclear factor-κB (NF-κB) and peroxisome proliferator-activated receptor (PPAR) to a certain degree. Collectively, the molecular perturbations driven by hyperglycemia seemingly demonstrate a tendency to converge on distinct genetic targets that are responsible for mediating vascular and neuronal injury in DR (10).

Certain key regulatory roles in the pathogenesis linked to DR have been ascribed to various genetic and epigenetic modulators, with miRNAs seemingly appearing to represent a particularly prominent category to a certain extent. Vascular hyperpermeability and neovascularization are induced through the process of hyperglycemia-driven VEGF upregulation and receptor activation, which in turn appears to modulate the expression profiles of various associated downstream genes and miRNAs (11–13). A range of miRNAs appear to show distinct functional involvement in the biological processes related to DR. Supplementation of miR-152, a molecule whose expression is reduced in human retinal vascular endothelial cells (hRVECs) that have been exposed to high glucose conditions appears to confer a protective effect through the process of reducing the expression of VEGF receptors (14). Similarly, reduced expression of miR-200 appears to be observed in both human hRVECs that have been challenged with high glucose and the retinal tissues obtained from diabetic rats. Targeted suppression of VEGF through the process of overexpressing miR-200 appears to yield inhibitory effects on vascular hyperpermeability and angiogenesis to a certain extent (15). Notably, the elevation of miR-200 levels also appears to decrease the expression of oxidation resistance 1 in Müller cells, a perturbation that may potentially impair the cellular protective capacity of these cells (16). In hRVECs, the induction of miR-146 that is driven by interleukin-1β (IL-1β) and TNF-α is abrogated by the inhibition of NF-κB activity. On the other hand, the administration of exogenous miR-146 exerts an attenuating effect on the activity of NF-κB, a phenomenon that appears to be indicative of a negative feedback regulatory loop (17). Within the experimental model system of oxygen-induced retinopathy, reduced expression of miR-126 exhibits a positive correlation with increased levels of VEGF, insulin-like growth factor 2 (IGF2), and hypoxia-inducible factor-1α (HIF-1α). The intravitreal administration of miR-126 seemingly mitigates these pathological phenotypes predominantly through the modulation of the p3-ERK signaling pathway (18, 19). An association with susceptibility to DR has also been established for a genetic variant (rs4636297) within a study cohort that comprises 531 patients (20). Further investigations have revealed that elevation of miR-29b levels in the retinal ganglion cells of STZ-induced diabetic rats may potentially exert anti-apoptotic effects via the PKR signaling axis and RNA-dependent protein kinase–associated protein X (21). Conversely, miR-195 appears to facilitate retinal endothelial damage through the suppression of SIRT1 expression (22). In contrast, miR-106a seems to confer protection against endothelial permeability elicited by high glucose via the downregulation of both HIF-1α and VEGF (23).

In recent years, noncoding RNAs (ncRNAs) have seemingly garnered increasing recognition as key modulators of gene expression and disease pathogenesis to a certain extent. Being devoid of the capacity for protein coding, these RNA species appear to exert regulatory control over gene activity at the post-transcriptional level. circRNAs have emerged as prominent members of this family alongside miRNAs and long noncoding RNAs (lncRNAs). Having been implicated in the process of cancer development, circRNAs have also been seemingly linked to DR in relevant research studies. From a structural perspective, circRNAs are observed to form covalently closed, continuous loop conformations through the process of back-splicing mechanisms. From a genomic perspective, four distinct subtypes are commonly recognized. These subtypes include exonic ones, which originate from either protein-coding or noncoding exons, intronic ones that are derived from retained introns, intergenic ones, and antisense ones that overlap untranslated regions in an antisense orientation (24, 25). Exonic circRNAs appear to be observed to frequently act as molecular sponges that sequester various associated miRNAs (26). A typical example can be found in ciRS-7, a brain-enriched circRNA that has more than around 70 conserved miR-7 binding motifs. Functional studies conducted in experimental settings seem to indicate that ciRS-7 sequesters miR-7, which may in turn potentially augment the expression of miR-7 target genes linked to certain malignancies and Alzheimer’s disease (27). In addition to the sequestration process of miRNAs, circRNAs also seem to show the potential ability to modulate the biological processes of mRNA splicing and protein translation (28).

Although extensive characterization studies and related work of circRNA functions have been carried out in the fields of cancer and various systemic disorders, their biological implications for different ocular pathologies appear to remain comparatively underexplored to some degree. Emerging research findings seem to indicate that circRNAs may potentially play a relevant role in the regulation of retinal function and vascular homeostasis in the clinical and experimental context of DR. Notable examples seem to include an inverse correlation between the progression of retinal fibrosis and the concentrations of circulating circRNAs (29), the observed association between various novel circRNA transcripts and atherosclerotic vascular disease (30), and the finding of hsa_circ_002059 as a potential diagnostic biomarker for gastric cancer, a designation that is based on its metastatic relevance (31). When taking into account the multifactorial pathogenesis linked to DR, which includes and involves various metabolic dysfunction processes, vascular damage events, and neuronal impairment mechanisms, along with the regulatory roles of miRNAs and lncRNAs that seemingly appear to have been fairly well-established in the progression associated with this ocular condition, the functional implications of circRNAs in this particular ocular disorder of DR appear to continue to be inadequately defined to a certain extent. To address this apparent critical knowledge void, the current investigation was designed in the process of systematically mapping circRNA expression profiles in the context of DR, with the aim of explaining the potential regulatory functions and possible diagnostic value of these noncoding RNA molecules.

## Methods

### Diabetic mice induced by STZ

Male C57BL/6 mice, weighing around 16–18 g, were obtained from Shanghai SLAC Laboratory Animal Co., Ltd. (SLAC, Shanghai, China). These associated experimental animals were housed under the condition of a 12-h light-dark cycle, with unrestricted access to standard laboratory chow and regular drinking water to some degree. The experimental protocol was reviewed and approved by the Ethics Committee of Tongji University, with all associated experimental procedures appearing to be conducted in strict adherence to institutional animal care regulations, as well as the guidelines set forth by ARVO and ARRIVE.

The mice were divided randomly into two groups: negative injection control (CT) and diabetic retinopathy (DR). For the purpose of inducing diabetes in the experimental mice, the animals were subjected to a 24-hour fasting period before they were administered an intraperitoneal injection of streptozotocin (STZ; Sigma, St. Louis, MO, USA). The STZ solution was freshly solubilized in a 0.05 M citrate buffer (pH 4.5) shortly before the injection was performed, and the dosage administered to each mouse was set at around 60 mg/kg of body weight. Diabetes was deemed verified to a certain extent when fasting blood glucose levels surpassed 300 mg/dL at 3 days following the STZ injection. Control mice were only injected with citrate buffer. For the purpose of capturing the early molecular perturbations that precede the emergence of overt retinal lesions, which seem to be consistent with the gradual progression of DR, analytical assessments were conducted at 6 weeks following the administration of STZ. A single intraperitoneal STZ injection seemingly yielded a diabetes induction success rate of 85%, with 34 out of the 40 injected mice developing the disease.

### Electroretinography

ERG assessments were carried out at 12 weeks post-STZ delivery, a specific time point that seemed to coincide with the manifestation of overt retinal functional deficits to some degree. Prior to the ERG recordings, the experimental mice were subjected to dark adaptation for a minimum duration of 12 consecutive hours. Anesthesia was initiated through the intraperitoneal injection of 1% sodium pentobarbital (administered at 0.1 mL per 20 g of body weight) in combination with xylazine hydrochloride (used at a 1:10 dilution, 0.01 mL/20 g of body weight; Huamu Pharmaceutical Co., Ltd., Jilin, China). Pupillary mydriasis was achieved using 0.5% tropicamide and phenylephrine (Santen Pharmaceutical Co., Ltd., Shanghai, China). Corneal anesthesia was administered via 0.5% proparacaine hydrochloride (Alcon-Couvreur NV, Puurs, Belgium) for the purpose of reducing potential discomfort during the experimental procedure.

The ERG detection system was composed of a corneal electrode, a reference electrode that was subcutaneously positioned at the anterior interocular scalp region, and a ground electrode that was implanted into the tail. Retinal response recordings were captured in the process of stimulation with three discrete flash intensities, specifically s1 = 6.325 × 10^-4^ cd·s/m², s2 = 6.325 × 10^-^³ cd·s/m², and s3 = 6.325 × 10^-^² cd·s/m² to a certain extent. Bilateral ocular evaluations were conducted for each experimental animal, with the control cohort comprising 15 mice and the DR group consisting of 12 mice. The reduced sample size in the DR cohort was attributable to anesthesia-associated incidents that occurred during ERG measurements, which seemingly hindered the completion of full assessment protocols in some of the treated animals to a certain degree.

### Fundus fluorescence angiography

Experimental animals were anesthetized following the protocol detailed previously, with the subsequent implementation of pupillary dilation and corneal anesthesia procedures to a certain extent. Fluorescein angiography was carried out through intraperitoneal injection of 10% fluorescein sodium (0.01 mL/20 g body weight; Baiyunshan Mingxing Pharmaceutical Co., Ltd., Guangzhou, China). Real-time fundus imaging acquisitions were conducted by making use of a Heidelberg Retina Angiograph (Heidelberg Engineering, Heidelberg, Germany). Microvascular pathological alterations were evaluated based on distinct angiographic manifestations, which seemingly included capillary nonperfusion areas, microaneurysms, and dye extravasation to a certain degree.

### Extraction of RNA

For the purpose of characterizing transcriptional perturbations that precede the onset of functional deficits, retinal tissues were dissected from both experimental cohorts (N = 15 animals per group) at six weeks post-STZ injection for the purpose of total RNA extraction. Absorbance measurements at the wavelength of 260 nm were carried out by making use of a NanoDrop ND-1000 spectrophotometer (Thermo Scientific, Waltham, MA, USA) to ascertain the purity and concentration of the isolated total RNA to a certain extent. Denaturing agarose gel electrophoresis was performed to evaluate the integrity of the extracted RNA. The activity of labeled complementary RNA (cRNA), a parameter that is defined as pmol dye per μg cRNA, was seemingly calculated via the following formula:


$$\text{Activity}\;=\;\left(\text{pmol}\;\text{dye}\;\cdot \;{\text{μL}}^{-1}\right)/\;\left(\text{μg}\;\text{cRNA}\;\cdot \;{\text{μL}}^{-1}\right)$$

### Microarray analysis of CircRNA

Enrichment of circRNA transcripts was seemingly achieved through the process of initial digestion of total RNA samples with RNase R (Epicentre, Inc.) to a certain extent. Subsequent amplification and fluorescent labeling of individual samples were carried out by making use of the Arraystar Super RNA Labeling Kit (Arraystar Inc.). Purification of the labeled cRNA was performed with the RNeasy Mini Kit (Qiagen), followed by the quantification of its concentration and activity with the help of a NanoDrop ND-1000 spectrophotometer. For the purpose of carrying out the hybridization procedure, 1 μg of the labeled cRNA was subjected to fragmentation in a solution that contains 5 μL of 10× blocking agent and 1 μL of 25× fragmentation reagent, with incubation carried out at the temperature of 60 °C for a duration of 30 minutes. After the process of fragmentation was completed, the resultant mixture was combined with 25 μL of 2× hybridization buffer for the purpose of yielding a final volume of 50 μL, which was then applied to Arraystar Mouse circRNA Arrays (8×15K, Arraystar). Hybridization was conducted at the temperature of 65 °C for a duration of 17 hours in a Agilent Hybridization Oven. Following the completion of hybridization, the microarray slides were subjected to the processes of washing and fixation, and subsequent scanning was performed using an Agilent Scanner G2505C.

Raw signal intensities derived from array images were seemingly obtained through the process of processing with Agilent Feature Extraction software to a certain extent. Subsequent data normalization was implemented by making use of the quantile approach, with downstream analytical procedures being conducted within the R software environment. Filtering of low-abundance circRNA transcripts was performed based on the criterion that each transcript must be detected, which was assigned a “p” or “m” flag, in a minimum of three out of six samples. Differential expression with statistical significance was potentially defined by two criteria: a fold change of approximately ≥2.0 or an adjusted p-value of ≤0.05 to a certain extent.

### CircRNA-miRNA network-targeted prediction and functional annotation

Potential circRNA-miRNA interaction pairs were seemingly predicted by making use of the proprietary Arraystar algorithm, which is observed to incorporate predictive outputs from TargetScan (32) and miRanda (33) to a certain extent. Computationally predicted miRNA-binding motifs were integrated into the annotation of differentially expressed circRNAs. Through this analytical process, 15 candidate miRNAs and 1, 261 putative target genes were seemingly identified and selected for the purpose of further investigation. GO term enrichment analysis was conducted on these target genes via miRWalk 2.0 (34), whereas comprehensive functional annotation was carried out using DAVID Bioinformatics Resources 6.7 (https://davidbioinformatics.nih.gov/home.jsp).

### Sequence conservation analysis and qPCR profiling

For the purpose of validating the qPCR findings, total RNA was seemingly subjected to reverse transcription into cDNA by making use of the SuperScript III Reverse Transcriptase system (Invitrogen, Shanghai, China) to a certain extent. Triplicate RT-PCR amplifications were carried out for each biological sample with the help of Arraystar PCR Master Mix, with GAPDH used as the endogenous reference gene. Fold changes in the expression levels were computed by means of the ΔΔCt method on the ViiA 7 RT-PCR System (Applied Biosystems), a process that seemingly yielded consistent results for the most part. Additionally, the nucleotide sequences of the characterized circRNAs were subjected to cross-species alignment between human and mouse genomes for the purpose of evaluating their potential evolutionary conservation to a certain degree.

### Statistical method-driven analysis

Data were seemingly presented as means ± standard deviation (SD) to a certain extent. The Shapiro-Wilk test was employed to evaluate the normality of the data distribution. Differences between the two experimental groups were analyzed by making use of an unpaired two-tailed Student’s t-test. Statistical significance was seemingly defined as a p < 0.05.

## Results

### Retinal function and STZ-induced diabetic mice-targeted assessment

#### DR model-targeted confirmation

**Figure 1:**
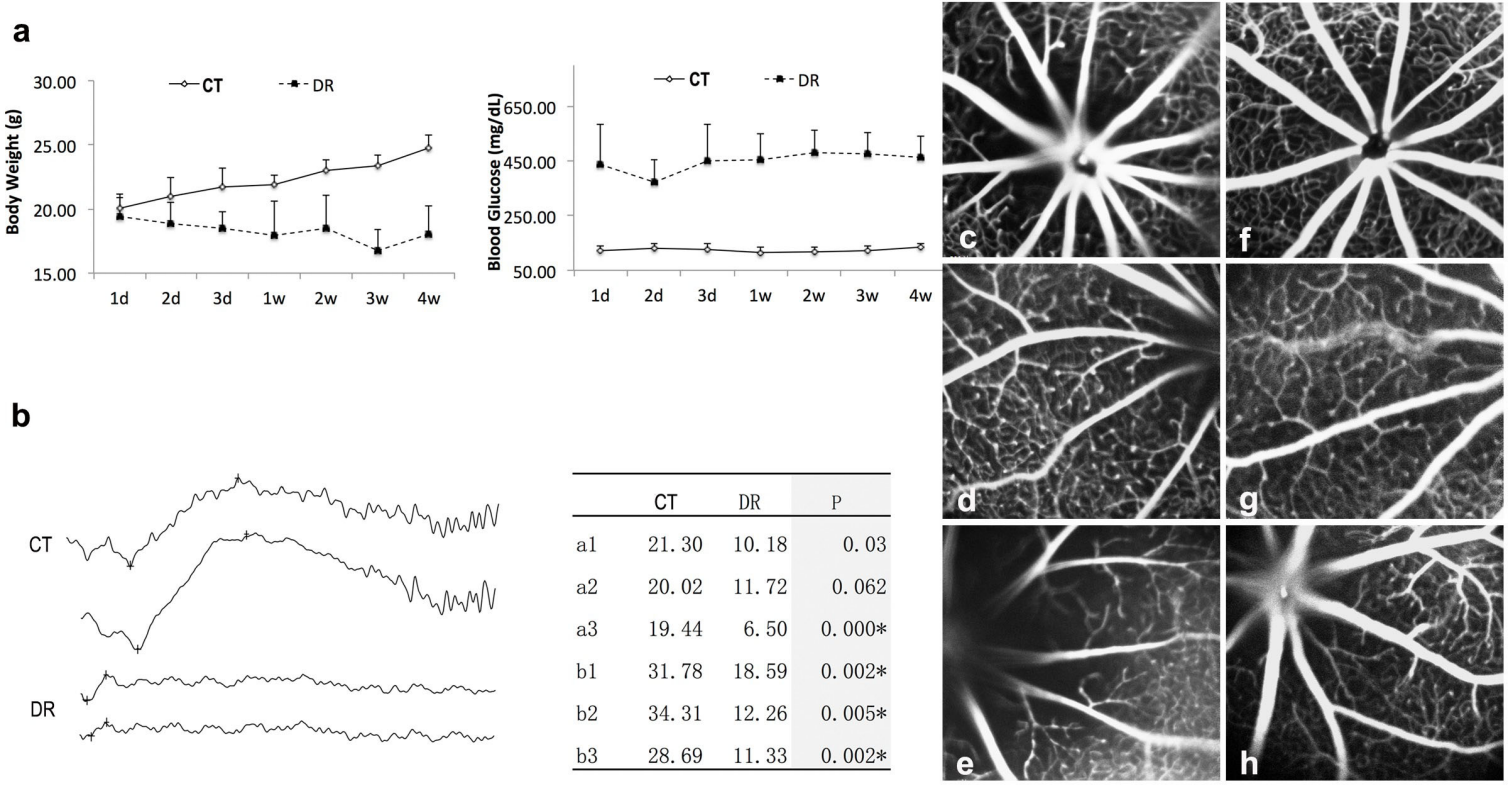
Body weight, blood glucose, ERG, and FFA examination of mice. **(a)** Body weight of mice was 17.99 ± 2.23 g vs. 24.7 ± 1.03 g in the DR group compared with the CT group at 4 weeks (*p<0.05); Blood glucose of mice was 462.5 ± 76.65 mg/dL vs 134.8 ± 12.25mg/dL in DR group compared with the CT group at 4 weeks (*p<0.05). **(b)** Typical ERG graphs and statistical analysis: there was no significant difference in a wave of ERG in lower flashlight level between control and DR groups (p>0.05); but both a wave (highest flashlight level) and b wave in the DR group were significantly decreased compared to that in the control group (*p<0.05). **(c-e)** Control group: uniform filling of fluorescence was seen in both retinal artery and veins, the capillaries were clear, straight and with equal diameter; **(f-h)** DR group: significant microvascular abnormities were observed, including hyper fluorescence dots and lower perfusion of fluorescence (10/10 mice).

Confirmation of the STZ-induced diabetic model was seemingly accomplished through the process of monitoring body weight and blood glucose concentrations to a certain extent. At four weeks post-induction, mice in the DR cohort were observed to exhibit a statistically significant reduction in body weight (17.99 ± 2.23 g) relative to control animals (24.79 ± 1.03 g; *p<0.05; **Figure 1a**), a difference that appeared to be biologically meaningful to a certain degree. Concurrently, blood glucose concentrations in DR mice were found to remain substantially elevated (462.5 ± 76.65 mg/dL) when compared to normoglycemic controls (134.8 ± 12.25 mg/dL; *p<0.05; **Figure 1a**), with this hyperglycemic state seemingly persisting consistently over the observation period.

#### Retinal functional- and structural-targeted assessment

At 12 weeks after the induction of diabetes in the experimental mice, assessments related to retinal function and vascular integrity were carried out through the use of ERG and FFA for the purpose of evaluating these key physiological parameters. Representative ERG tracings were observed to seemingly suggest diminished a-wave and b-wave amplitudes in the DR mouse cohort to a certain extent (**Figure 1b**). Quantitative analytical assessments were found to reveal no statistically significant variation in a-wave amplitude under lower flash intensity conditions (p>0.05). The DR cohort, however, was observed to show a statistically significant reduction in both the a-wave amplitude under the maximum stimulus intensity conditions and the b-wave amplitudes (*p<0.05; **Figure 1b**). FFA imaging obtained from control animals was observed to display balanced arterial and venous fluorescence alongside well-demarcated capillary networks (**Figures 1c-e**). In contrast, DR retinas were found to exhibit prominent microvascular pathological changes, which seemingly were characterized by hyperfluorescent foci and areas of capillary nonperfusion to a certain extent (**Figures 1f-h**). These vascular perturbations were detected in all of the evaluated DR mouse subjects (N = 10), an experimental finding that appeared to potentially suggest their possible correlation with the diabetic condition to a certain extent.

### Differential expression analysis coupled with CircRNA microarray profiling

Microarray profiling was carried out on retinal tissues that were harvested at 6 weeks post-induction for the purpose of clarifying DR-associated circRNA expression perturbations to a certain extent. RNA quality was validated through A260/A280 ratios that spanned approximately 1.87 to 1.96, with this validation being complemented by distinct 28S and 18S ribosomal RNA bands that were observed via the process of denaturing agarose gel electrophoresis. The activity of the labeled cRNA was found to potentially span approximately 13.62-17.12 pmol dye per μg cRNA.

**Figure 2:**
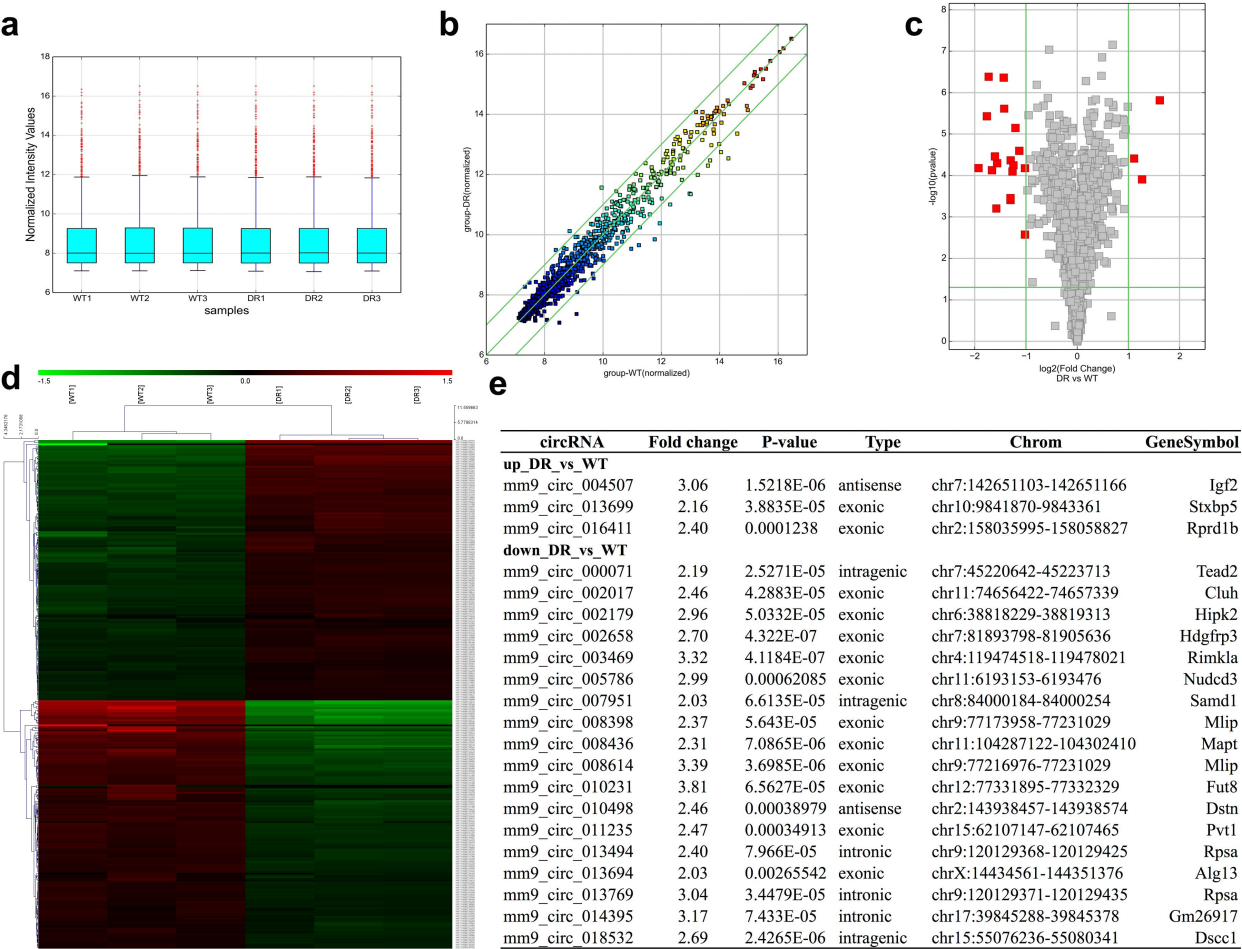
Quantification of circRNA microarray analysis and differentially expressed circRNAs between nondiabetic and diabetic retinas. **(a)** Boxplot view provides a comparison of the distributions of expression values for the 6 samples after normalization. **(b)** The scatterplot shows the CircRNA expression variation between the two compared groups. The green lines represent fold change. The circRNAs above the top green line and below the bottom green line indicated more than two-fold change of circRNAs between two samples. **(c)** Volcano plot shows that there were several red points that were outside the vertical lines (correspond to two-fold up/down) and the horizontal line (represent a value of p = 0.05). **(d)** The results of hierarchical clustering showed distinguishable circRNA expression profiling among samples and arrangements based on their expression levels. **(e)** There were 3 upregulated and 18 downregulated differentially expressed circRNAs (named according to the circBase); the fold changes were arranged from 2.03 to 3.81.

In the process of carrying out data normalization, a box plot was generated for the purpose of illustrating the distribution of expression profiles across the six associated experimental samples, which were presented in the form of log_2_-transformed raw signal ratios (**Figure 2a**). A scatter plot was constructed to visualize the global expression differences between the control (CT) and DR experimental cohorts, with the axes of the constructed plot corresponding to the average normalized signal intensity (on a log_2_ scale) of each respective experimental group (**Figure 2b**). To further characterize the differentially expressed circRNAs, a volcano plot, which plots fold change versus p-value, was generated, where red dots were observed to seemingly denote transcripts that satisfied the set criteria of a 2-fold change and p≥0.05 (**Figure 2c**) to a certain extent. Nested clustering analysis of all circRNA targets was found to seemingly reveal distinct expression profiles among the experimental samples that were clustered based on the similarity in their respective expression levels (**Figure 2d**). Subsequent analysis seemingly identified 3 upregulated and 18 downregulated circRNAs, which were annotated with circBase identifiers, with fold changes that spanned approximately 2.03 to 3.81. These differentially expressed transcripts were classified into four primary genomic subtypes, namely antisense, exonic, intronic, and intragenic (**Figure 2e**), a categorization that appeared to be consistent with the classifications reported in previous studies.

### Profiling of differential circRNA expression via microarray analysis

**Figure 3:**
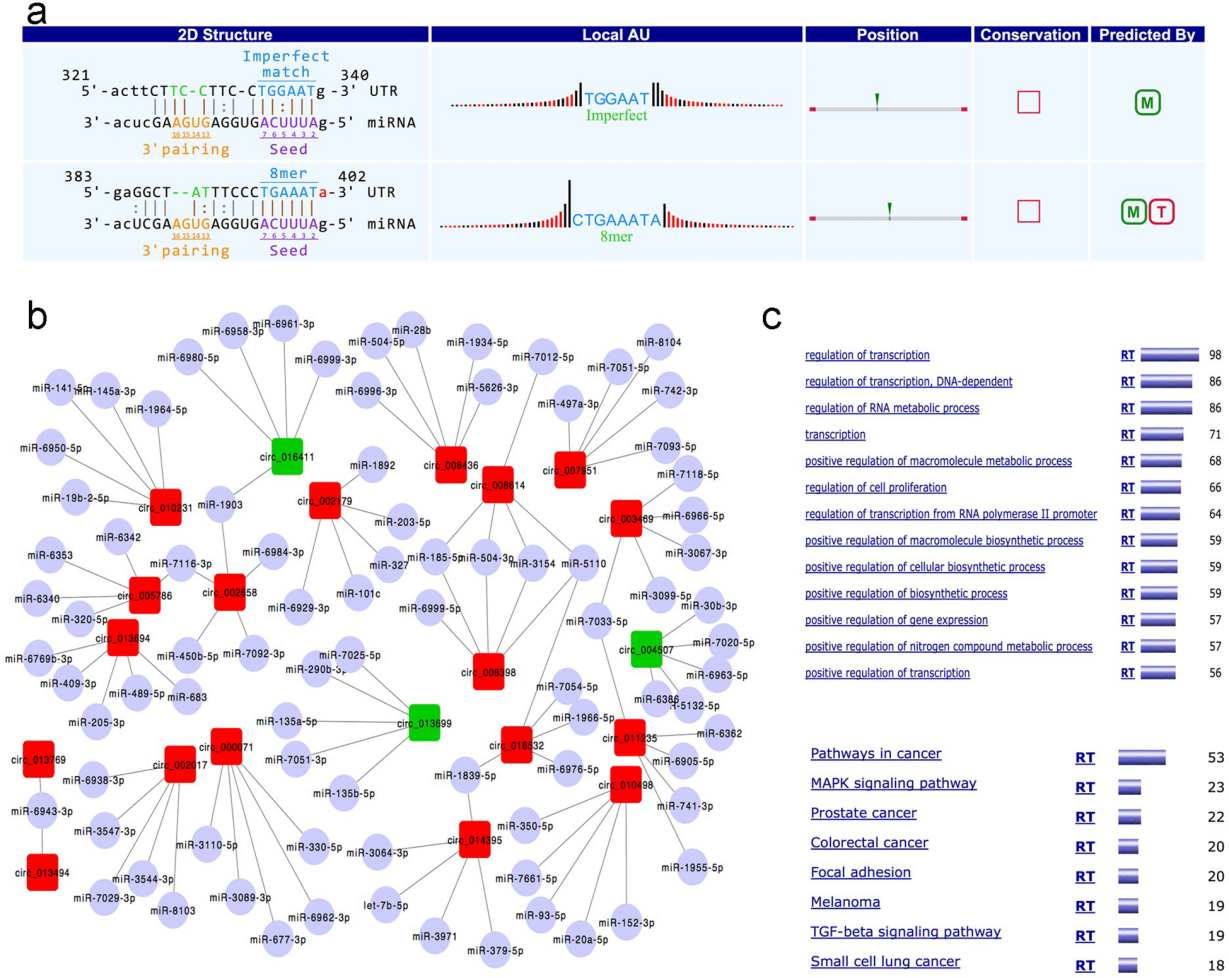
circRNA-miRNA interaction and functional annotation. **(a)** Example of complementarity and little variation binding based on prediction of circRNA-microRNA interaction. **(b)** 3 upregulated and 18 downregulated circRNAs and their targeted microRNAs, and several overlapping targeted microRNA were observed. **(c)** GO function studies of circRNA-miRNA targeted genes showed the most important function and the highest enriched processes of circRNA.

CircRNAs have been documented to function as miRNA molecular sponges through the process of miRNA sequestration, thereby potentially exerting competitive inhibition on miRNA activity and regulating their downstream regulatory roles to a certain extent. To explore this regulatory mechanism, computational predictions were carried out to identify potential circRNA-miRNA interaction pairs, with the concurrent annotation of all differentially expressed circRNAs. **Figure 3a** illustrates a representative predicted interaction, which is characterized by complementary base-pairing and low binding variability to a certain degree. Subsequently, the co-expression network constructed for the 18 downregulated and 3 upregulated circRNAs alongside their corresponding target miRNAs was found to uncover multiple miRNAs that are shared among several circRNAs (**Figure 3b**). Network analysis revealed that multiple miRNAs were commonly targeted by several circRNAs, suggesting the presence of shared regulatory nodes within the circRNA-miRNA interaction landscape. Leveraging the predicted miRNA targeting profiles, downstream mRNA targets of these miRNAs were compiled for the purpose of GO functional enrichment analysis. miRWalk 2.0 (34) was employed to identify 15 candidate miRNAs and 1, 261 associated target genes, a process that seemingly yielded reliable results. GO enrichment analysis was observed to demonstrate that the predominant functional categories centered on transcriptional regulation, with the most significantly enriched pathways linked to cancer (**Figure 3c**), indicating that the identified circRNA-miRNA-mRNA network may be involved in key biological processes such as cell proliferation, apoptosis, and angiogenesis.

### CircRNA conservation analysis coupled with PCR confirmation​

**Figure 4:**
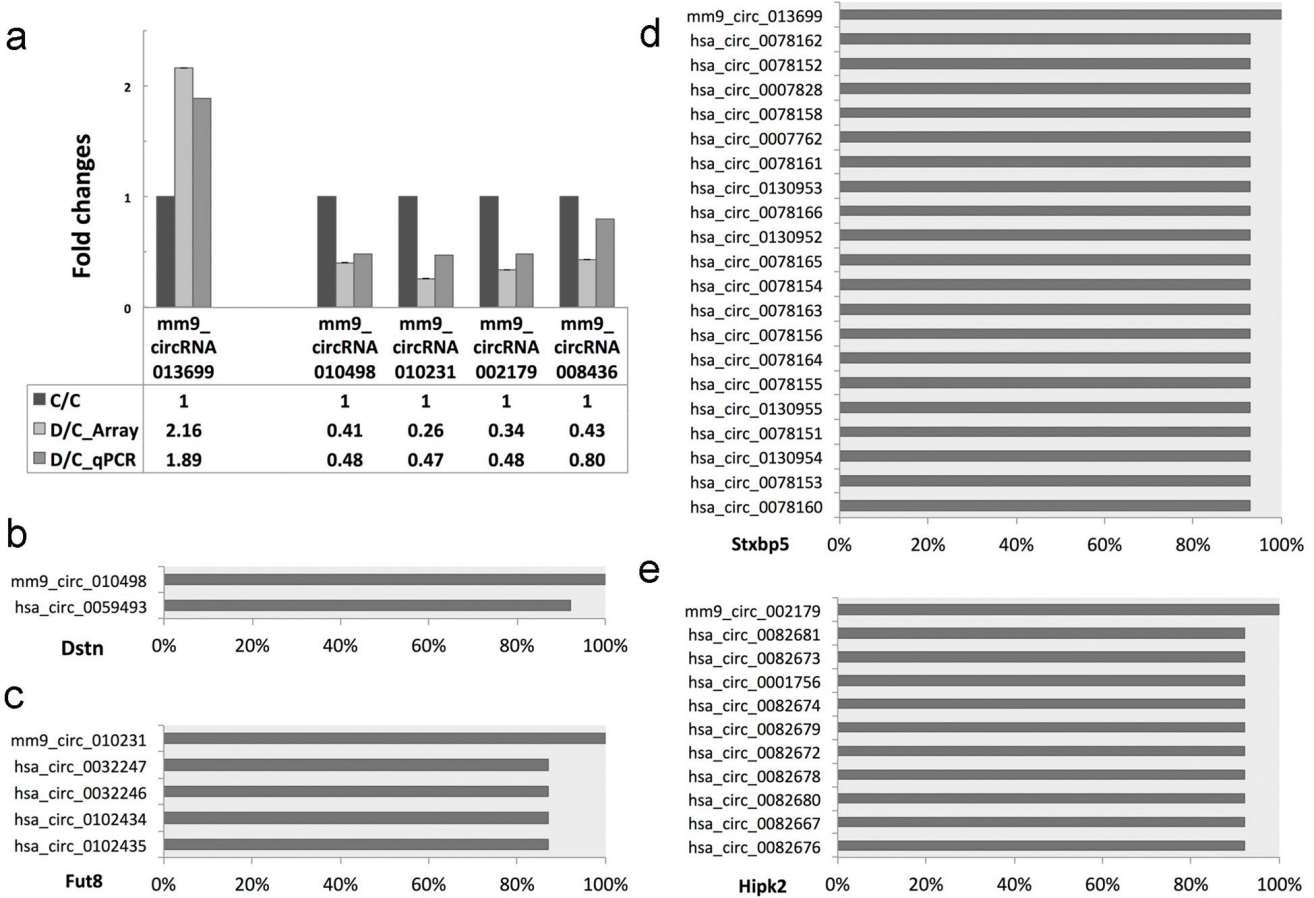
PCR confirmation and conservation of circRNAs. **(a)** qPCR of circRNA: The fold change of upregulated mm9-circRNA-013699 expression was 1.89 vs 2.16 by qPCR compared to microarray. The fold changes of decreased expression of downregulated mm9-circRNA-010498, mm9-circRNA-010231, mm9-circRNA-002179, and mm9-circRNA-008436 were 0.41, 0.26, 0.34, and 0.43 vs 0.48, 0.47, 0.48, and 0.80 by qPCR compared to microarray. **(b–e)** Blast result between human circRNA and mouse circRNA: there were 1, 4, 20, and 10 human circRNAs with high similarity to mice mm9-circRNA-013699, mm9-circRNA-010498, mm9-circRNA-010231, and mm9-circRNA-002179, respectively.

qPCR validation was carried out on five circRNAs that exhibit dysregulated expression patterns to a certain extent. For the upregulated circRNA mm9-circRNA-013699, qPCR was observed to yield a fold change of 1.89, a value that appeared to be consistent with the microarray-derived fold change of 2.16. The four downregulated circRNAs, mm9-circRNA-010498, mm9-circRNA-010231, mm9-circRNA-002179, and mm9-circRNA-008436, were found to display qPCR-determined fold changes of 0.41, 0.26, 0.34, and 0.43, respectively, which seemed to be consistent with their corresponding microarray fold changes of 0.48, 0.47, 0.48, and 0.80 (**Figure 4a**). To further assess their evolutionary conservation, sequence homology analysis was conducted across the human genome. The results indicated a high degree of conservation among the selected circRNAs. Specifically, BLAST alignment identified 1, 4, 20, and 10 human circRNA homologs with high sequence similarity to the murine circRNAs mm9-circRNA-013699, mm9-circRNA- 010498, mm9-circRNA-010231, and mm9-circRNA-002179, respectively (**Figures 4b-e**).

## Discussion

circRNAs are increasingly recognized as pivotal functional regulators in diverse biological processes and disease pathogenesis. In this study, microarray profiling of retinal tissues from STZ-induced diabetic mice identified 21 differentially expressed circRNAs, including 3 upregulated and 18 downregulated transcripts, which were classified into four genomic subtypes: antisense, exonic, intronic, and intragenic. A well-characterized functional role of circRNAs was acting as endogenous miRNA molecular sponges. Consistent with this functional paradigm, our analyses revealed a relatively complex regulatory network in which multiple miRNAs are co-targeted by several distinct types of circRNAs, in which multiple circRNAs converge on shared miRNAs, suggesting coordinated regulation of miRNA activity. The predominance of downregulated circRNAs may lead to enhanced miRNA-mediated repression of target genes, thereby contributing to transcriptional dysregulation. These findings suggest that circRNA-miRNA regulatory networks may play a role in the pathogenesis of diabetic retinopathy. However, further experimental validation is required to confirm these interactions and clarify their functional significance.

A typical example of such circRNA-miRNA crosstalk is ciRS-7, a highly conserved circRNA enriched in mammalian brain tissue that has been widely reported in studies to potentially bind and sequester miR-7, thereby seemingly serving to de-repress the expression of miR-7 target genes associated with various pathological processes to a certain extent. Similarly, the analysis carried out in the present study appeared to show distinct miRNA regulatory networks linked to the expression-altered circRNAs in the retinas of DR mice, with the potential relevance of this regulatory axis to retinal pathology being further highlighted in the process. Specific GO enrichment analysis of the predicted miRNA-targeted genes further appeared to reveal a seemingly significant overrepresentation of pathways related to transcriptional regulation and cancer-associated signaling. These results not only corroborate the established role of circRNAs in modulating transcriptional programs but also suggest their broader involvement in disease-relevant regulatory networks. Collectively, this study expands the current understanding of circRNA-miRNA interactions by implicating their potential contribution to the molecular mechanisms underlying DR. Numerous enriched cancer-related pathways seem to potentially include and involve various core biological processes that are implicated in the pathogenesis of DR, including apoptosis, angiogenesis, and cell proliferation. DR is characterized by early disruption of the inner blood-retinal barrier and apoptosis of retinal neuronal cells, followed by the occurrence of pathological neovascularization during the proliferative phase of the condition. These biological processes appear to mirror the cellular and molecular alterations that drive tumor progression. This functional overlap appears to suggest that circRNA-miRNA-regulated genes may potentially constitute key drivers in the pathological progression process of DR. Notably, several of the identified target genes are associated with the pathway of focal adhesion signaling, a specific pathway that is critical for the maintenance of blood-retinal barrier integrity through the process of regulating endothelial cell adhesion and junction stability. Furthermore, the enrichment of cytokine receptor-related genes implicates circRNA-miRNA networks in the modulation of inflammatory pathways. These inflammatory pathways are well-documented to contribute to DR pathology by exacerbating vascular dysfunction and neuronal damage to some degree. Importantly, these findings suggest that targeting circRNA–miRNA regulatory axes may represent a promising therapeutic strategy for the prevention or treatment of DR.

qPCR validation of five distinct circRNAs confirmed the accuracy of the initial microarray findings. Sequence alignment analysis revealed a high degree of conservation between the mouse circRNAs and their corresponding human homologs, which seemingly indicates that the identified circRNAs may have conserved biological functions. Notably, our experimental findings appear to reflect the initial molecular alterations occurring in the context of DR, with the expression dynamics of these circRNAs potentially exhibiting variations over a prolonged duration of diabetic condition. This experimental study appears to have certain inherent limitations: on one hand, microarray analysis may not be capable of detecting all circRNA isoforms that exist in the retinal tissues; on the other hand, no *in vivo* functional validation was carried out for the circRNAs identified in the present investigation. Therefore, further studies incorporating functional experiments and *in vivo* models are required to validate these findings and elucidate the precise roles of circRNAs in DR.

Recent studies have increasingly demonstrated that circRNAs play critical regulatory roles in the pathogenesis of DR, particularly through functioning as miRNA sponges to modulate retinal vascular endothelial cell behavior. Specifically, circHIPK3, which is predominantly localized in the cytoplasm of HVECs, is also markedly upregulated. By suppressing miR-30a-3p activity, circHIPK3 promotes the expression of downstream targets, including VEGFC, FZD4, and WNT2, thereby facilitating abnormal endothelial cell proliferation, migration, and tube formation. Notably, knockdown of circHIPK3 effectively attenuates these pathological changes, reduces the formation of acellular capillaries, and alleviates vascular leakage. These findings indicate that circHIPK3 may play a crucial role in the progression of non-proliferative diabetic retinopathy (NPDR) (35). This observation further highlights the role of circRNAs in regulating the balance between cell survival and death during DR progression. Collectively, these findings suggest that circRNAs contribute to DR pathogenesis by forming circRNA-miRNA-mRNA regulatory networks that govern key endothelial cell processes, including proliferation, migration, apoptosis, and vascular permeability. These molecules may serve as potential therapeutic targets for DR; however, the precise molecular mechanisms underlying their effects warrant further investigation.

## Conclusions

Three specifically upregulated and 18 specifically downregulated conserved circRNAs were identified in the retinal tissues of diabetic mice in the present study. It is hypothesized that these circRNAs may potentially contribute to the pathogenesis of DR by acting as relevant miRNA sponges, thereby potentially modulating the processes of vascular integrity maintenance, neuronal function regulation, and inflammatory signaling pathway activation to some degree. qPCR validation was conducted on five representative circRNAs, including one specifically upregulated and four specifically downregulated transcripts, with the results seemingly corroborating the accuracy of the initial microarray profiling findings to a certain extent. Meanwhile, the cross-species sequence alignment analysis carried out in the present study was found to seemingly reveal a high degree of evolutionary conservation between the murine circRNAs and their respective human homologs, which appeared to potentially support the potential translational relevance of these experimental findings for the field of clinical DR research to a certain extent.

The present study was found to have certain inherent limitations that appear to call for explicit recognition to a certain extent. First, the microarray-based detection method was found to have a certain inherent constraint, in that it cannot capture the full spectrum of circRNA isoforms, particularly those isoforms with low expression abundance or sequences that are not represented in the corresponding array probe library. Second, *in vivo* functional validation of the identified circRNAs, including verification of their miRNA sponging activity and direct regulatory roles in retinal pathophysiology, remains pending. Further investigations are required to elucidate the precise biological functions of these conserved circRNAs, such as their direct miRNA targets and the downstream signaling cascades they modulate. Such studies hold promise for uncovering novel therapeutic opportunities for DR by targeting the circRNA-miRNA regulatory network.

## Data Availability

The original contributions presented in the study are included in the article/supplementary material. Further inquiries can be directed to the corresponding author.
